# Combined exposure to multiple essential elements and cadmium at early pregnancy on gestational diabetes mellitus: a prospective cohort study

**DOI:** 10.3389/fnut.2023.1278617

**Published:** 2023-12-06

**Authors:** Guifang Deng, Hengying Chen, Yao Liu, Yingyu Zhou, Xiaoping Lin, Yuanhuan Wei, Ruifang Sun, Zheqing Zhang, Zhenhe Huang

**Affiliations:** ^1^Department of Clinical Nutrition, Union Shenzhen Hospital of Huazhong University of Science and Technology, Shenzhen, China; ^2^Department of Maternal and Child Health, School of Public Health, Tongji Medical College, Huazhong University of Science and Technology, Wuhan, China; ^3^Guangdong Provincial Key Laboratory of Food, Nutrition and Health, Guangzhou, China; ^4^Department of Nutrition and Food Hygiene, Guangdong Provincial Key Laboratory of Tropical Disease Research, School of Public Health, Southern Medical University, Guangzhou, China; ^5^Geriatric Medicine Department, Union Shenzhen Hospital of Huazhong University of Science and Technology, Shenzhen, China

**Keywords:** elements, gestational diabetes mellitus, mixed exposure, zinc, copper, cadmium

## Abstract

**Background:**

Minerals and trace elements were involved in the pathogenesis and progression of diabetes. However, the association of mixed exposure to essential elements and toxic elements with gestational diabetes mellitus (GDM) is poorly understood.

**Objective:**

This study aims to examine the associations between serum calcium (Ca), iron (Fe), zinc (Zn), copper (Cu), magnesium (Mg), and cadmium (Cd) concentrations in early pregnancy and GDM risk in Chinese pregnant women.

**Method:**

A total of 1,168 pregnant women were included in this prospective cohort study. The concentrations of serum elements were measured using the polarography method before 14 gestational weeks and an oral glucose tolerance test was conducted at 24–28 gestational weeks to diagnose GDM. Binary logistic regression analysis and restricted cubic spline were applied to evaluate the association between serum individual element and GDM. Bayesian kernel machine regression (BKMR) and weighted quantile sum (WQS) regression were used to assess the associations between mixed essential elements and Cd exposure and GDM risk.

**Results:**

The mean concentrations of Zn (124.65 vs. 120.12 μmol/L), Fe (135.26 vs. 132.21 μmol/L) and Cu (23.33 vs. 23.03 μmol/L) in the GDM group were significantly higher than those in the control group. Single-element modeling results suggested that second and fourth-quartile maternal Zn and Fe concentration, third and fourth-quartile Cu concentration and fourth-quartile Ca concentration were associated with an increased risk of GDM compared to first-quartile values. Restricted cubic spline analysis showed U-shaped and non-linear relationships between Cd and GDM. According to the BKMR models and WQS analyses, a six-element mixture was significantly and positively associated with the risk of GDM. Additionally, Cd, Zn, and Cu contributed the most strongly to the association.

**Conclusion:**

Serum Zn, Cu, Fe, and Ca exposure during early pregnancy showed a positive association with GDM in the individual evaluation. The multiple-evaluation showed that high levels of elements mixture, particularly Cd, Zn, and Cu, may promote the development of GDM.

## 1 Introduction

Gestational diabetes mellitus (GDM) is characterized by poor glucose tolerance, which occurs or is first discovered during pregnancy and affects 9%–26% of pregnant women worldwide (1, 2). The latest available data showed that the total incidence of GDM in mainland China ranged from 12.8% to 16.7%, which raised growing public health concerns (3). The negative health effects of GDM on mothers and infants have been well established (4). To date, most diagnostic criteria guidelines for GDM recommend the oral glucose tolerance test (OGTT) during gestationally 24–28 weeks, leading to a narrow therapeutic window (5, 6). However, evidence of identified effects was provided from the experimental intervention that was applied to prevent GDM in the first trimester of pregnancy, reinforcing the need for biomarkers in early pregnancy.

Minerals and trace elements were considered to play specific roles in the pathogenesis and progression of diabetes. Increasing animal and metabolic studies demonstrated that several essential elements, including zinc (Zn), iron (Fe), manganese (Mg), copper (Cu), and calcium (Ca), can affect glucose metabolism and insulin sensitivity with downstream effects on hyperglycemia and GDM (7, 8). For example, Zn is involved in the synthesis and secretion of insulin from the pancreatic beta-cells (9), while Mg-containing enzymes are involved in glucose homeostasis (10). Previous epidemiologic studies mainly focused on heavy metals (11–14), while only a few studies that have investigated the effects of essential elements on the risk of GDM have yielded inconsistent results (15, 16). A nested case-control study with 305 pairs of GDM and controls showed that the increase of Cu concentration was positively correlated with the risk of GDM, and Zn had a negative impact on GDM (17). However, there is no association between Zn and Cu and GDM (16, 18), and even opposite results have been reported in other studies (15). Recently, a retrospective study from South China indicated that serum Mg and Ca in the first trimester were significantly associated with fasting plasma glucose during mid-pregnancy (15), but these findings are not supported by the studies of others (19, 20).

It has also been shown that elevated heavy metal levels, such as cadmium (Cd), can impair insulin secretion by inducing damage to pancreatic islet b-cells through oxidative stress (21). A number of epidemiologic studies have evaluated Cd as it relates to GDM (12, 22, 23). It has been revealed that many toxic effects of cadmium (Cd) action result from interactions with essential elements, including Zn, Fe, and Ca (24). However, in most population-based studies, only a single effect of individual essential elements on GDM has been evaluated. In the real world, pregnancies are exposed to both essential and heavy elements rather than one single element or one class of elements, thus investigating the relationship between the mixed essential and heavy elements and GDM is also required. Due to the multicollinearity and mixture among elements, the traditional logistic regression used in most previous studies may be biased and underestimate the actual risk in a “real world” situation (25).

Therefore, this prospective cohort study aims to use two novel statistical analysis models, Bayesian kernel machine regression (BKMR) and weighted quantile sum (WQS) regression, to investigate the association between essential serum elements (Mg, Fe, Cu, Zn, and Ca) plus Cd during the first trimester and GDM. These statistical analysis models have already been applied to untangle the combined effects of mixed elements on other health conditions (e.g., miscarriage and allergy) (26, 27).

## 2 Materials and methods

### 2.1 Study design and population

This study was based on a prospective cohort conducted at Huazhong University of Science and Technology Union Shenzhen Hospital in Shenzhen, China (registration number: ChiCTR2200056287). Pregnant women residing in Shenzhen city and coming for their first prenatal care visit (before 12 weeks) were invited to participate in the study, with the willingness to provide blood samples at 24–28 weeks of pregnancy, 32–37 weeks of pregnancy and 42 days of postpartum and to complete a face-to-face interview questionnaire. At baseline, a total of 6,201 pregnant women aged 20–45 years were enrolled from April 2019 to May 2021. According to our pre-specified inclusion and exclusion criteria of participants for the present analysis (Supplementary Figure S1), the pregnant women with preexisting type 1 or type 2 diabetes before pregnancy (*n* = 27), multiple pregnancies (*n* = 46), *in vitro* fertilization (*n* = 122), hepatitis (*n* = 149) and hyperthyroid (*n* = 66) were excluded. Further, we also excluded pregnancies with missing information on any serum elements (e.g., Fe et al.) at the first trimester (*n* = 4,080) and OGTT during gestational 24–28 weeks (*n* = 543), leaving 1,168 pregnant women included in the final analysis. Maternal demographic characteristics and pregnancy outcomes were compared between the mothers who were included in the analyses and the mothers who were excluded from the analyses. As a result, baseline maternal covariates and pregnancy outcomes were primarily similar between these two groups (Supplementary Table S1). Ethical approval for this cohort was given by the Ethics Committee of Huazhong University of Science and Technology Union Shenzhen Hospital (No. 2019072644).

### 2.2 Measurements of serum elements in the first trimester

Zn, Ca, Cu, Mg, Fe, and Cd concentrations were measured in blood serum samples collected during the first trimester of pregnancy (median: 9 weeks of gestation). The venous blood samples (3 mL) were collected by professional nurses in the morning and then transported to the laboratory within 1 h for measurement of serum elements concentration using the polarography method (LK98B, LANBIAO, Tianjin, China). Following a rigorous vortex mixing procedure, 40 μL of serum sample was transferred into the rotating cup of the vitamin analyzer and 2 mL of sample processing solution (analytically pure, LANBIAO) was supplemented. In each batch, we also processed and analyzed a blank sample, consisting solely of 1.2% HNO_3_, to monitor and control any potential contamination vigilantly. Notably, the spike recovery values for serum metals fell within the range of 98.25% to 105.66%. The detection limit of the polarographic channel was ≤1 × 10^−10^ mol/L, and measurements of six serum elements were above the detection limit for all of the samples. The average inter-day coefficients of variation (CV) for six serum elements range from 3.75% to 9.02%.

### 2.3 Assessment of GDM

All pregnant women in the current study underwent a routine 75 g oral glucose tolerance test at 24–28 gestational weeks to diagnose GDM. Serum glucose concentrations were measured enzymatically on a 7600–010 automated biochemical analyzer (Hitachi, Tokyo, Japan). In accordance with the International Association of Diabetes and Pregnancy Study Group (IADPSG) criteria, GDM was diagnosed if any of the following conditions were met: the fasting plasma glucose was >5.1 mmol/L and/or postprandial blood glucose at 1 h was >10.0 mmol/L and/or postprandial blood glucose at 2 h was >8.5 mmol/L (5).

### 2.4 Covariates

Socio-demographic information collected at baseline via standard questionnaires by face-to-face interview included: household registration (local resident or temporary resident), maternal education level (junior middle school and below, high school or university and above), family history of diabetes (at least one of the immediate family members had type 1 diabetes or type 2 diabetes; Yes or no), previous history of diabetes (yes or no), gravidity (one time or ≥2 times), history of abortion (yes or no), maternal age (continuous) and pre-pregnancy body weight (continuous). Height was measured without shoes to the nearest 0.1 cm by trained nurses. Pre-pregnancy body mass index was calculated as pre-pregnancy body weight (kg) divided by height squared (m^2^). Fetal gender (male or female), parity (one time or ≥2 times), gestational age at delivery, birth weight and birth height were derived from the maternal delivery records. *A priori*, the above variables except pregnancy outcomes were selected as potential confounders based on previous literature (28–30). We also constructed a directed acyclic graph (DAG; Supplementary Figure S2) to retain a minimally sufficient number of confounders in the regression models (31). The following confounders were identified as being important: maternal age, maternal education level, pre-pregnancy BMI and self-reported history of diabetes.

### 2.5 Statistical analysis

Characteristics of the study population were described as means and standard deviations (SD) for continuous variables and frequencies, as well as the frequency and percentage of categorical variables. Differences across groups with or without GDM were tested using t-tests, *χ*^2^ tests or Fisher’s exact test when appropriate. Serum element concentrations were natural log (ln) transformed due to the positively skewed distribution. In addition, Pearson’s correlation analysis was used to explore the correlations between the levels of six elements in serum. The multivariate imputation by chained equations (MICE) method was carried out to account for missing values for a small proportion of missing pre-pregnancy BMI (1.80%) and parity (0.17%) values. A total of 5 imputations were performed separately, and the imputed datasets were pooled together as a single dataset with mean values for pre-pregnancy BMI and mode values for parity.

Our statistical analysis consists of four parts. First, the concentrations of the serum element were divided into four categories according to the quartile they fell into, and the lowest group was used as the reference. A binary logistic regression model was fit to investigate the dose-response associations between individual element concentration and GDM, including (1) model 1: without any adjustment and (2) model 2: adjusted for a minimally sufficient set of confounders (maternal age, maternal education level, pre-pregnancy BMI, and self-reported history of diabetes) identified by DAG. Tests for linear trends were performed using the median concentrations of elements in each quartile as a continuous variable.

Second, a restricted cubic spline regression with three knots at 25th, 50th and 75th was used to evaluate the possible nonlinear relationship between the levels of serum elements and GDM. Third, the weighted quantile sum (WQS) regression model was applied to evaluate the joint effect of elements. All elements are considered in this approach, and elements included in this model were restricted to have the same effect direction for the association. The WQS index that reflects the body burden of element mixtures and the weight that suggests the contribution of each element were calculated, respectively. Two sets (positive and negative) of WQS regression models in relation to GDM were conducted, each of which derived a WQS index by bootstrapping 10,000 times separately. A similar procedure has been used in several epidemiologic studies (32, 33).

Finally, the probit extension of Bayesian kernel machine regression (BKMR) was used to flexibly model the adjusted association of the element mixture at the first trimester on the risk of GDM with consideration of the possible nonlinear and non-additive associations between element mixtures and GDM. We conducted a component-wise variable selection method with 10,000 iterations by a Markov chain Monte Carlo (MCMC) algorithm and estimated the posterior inclusion probability (PIP) that reveals the relative importance of each element exposure for selecting crucial elements. Higher values indicate higher importance, and the threshold value of PIP was set at 0.5 (34). The covariates adjusted for in spline analyses, WQS and BKMR models were similar to model 2 in the multivariate logistic regression analysis.

In order to verify the robustness of our main results, two sensitivity analyses were performed: firstly, a model with adjustment for the full set of confounders because these factors have been proved to either affect the levels of elements, the glucose level or the risk of GDM. Secondly, to address potential bias resulting from the weak correlation observed between Cd and other elements, we selected the quantile g-computation model as an alternative analysis method. This approach is renowned for its impartiality, even when dealing with decreased correlations between the two exposures, and was used to replicate our findings (35). All the analyses were performed using R statistical software (version 4.2.1, http://www.r-project.org). For all analyses, a significant *p*-value (two-tailed) was defined as *p* < 0.05.

## 3 Results

A total of 1,168 women were included in this study, of whom 383 (32.79%) were diagnosed with GDM at 24–28 weeks of gestation (Table 1). Compared to pregnant women without GDM, GDM women were more likely to be primiparous (*p* = 0.04) and have a family history of diabetes (*p <* 0.01). They also tend to be older (*p* = 0.02) and have a higher pre-pregnancy BMI (*p* < 0.01) and a lower gestational age at delivery (*p* = 0.03). There were no significant differences between the GDM and non-GDM with respect to key maternal and fetal characteristics: household registration, education level, self-reported history of diabetes, gravidity, history of abortion, fetal gender, birth weight and birth height (all *p* > 0.05).

**Table 1:** Descriptive characteristics of the study population.

Characteristics	GDM, *N* = 383	Non-GDM, *N* = 785	*p*-value^a^
Maternal age, years	32.77 ± 4.20	32.15 ± 3.78	0.02
Pre-pregnancy BMI, kg/m^2^	21.96 ± 2.78	20.60 ± 3.74	<0.01
Household registrations, *n* (%)			0.15
Local resident	249 (65.01)	543 (69.17)	
Temporary resident	134 (34.99)	242 (30.83)	
Maternal education, *n* (%)			0.36
Junior middle school and below	15 (3.92)	27 (3.44)	
High school	159 (41.51)	295 (37.58)	
University and above	209 (54.57)	463 (58.98)	
Family history of diabetes, *n* (%)			<0.01
Yes	7 (1.83)	0 (0.00)	
No	376 (98.17)	785 (100.00)	
Self-reported history of diabetes, *n* (%)			0.08
Yes	8 (2.09)	6 (0.76)	
No	375 (97.91)	779 (99.24)	
Gravidity, *n* (%)			0.93
1	100 (26.11)	203 (25.86)	
≥2	283 (73.89)	582 (74.14)	
Parity, *n* (%)			0.04
1	170 (44.39)	300 (38.22)	
≥2	213 (55.61)	485 (61.78)	
History of abortion, *n* (%)			0.54
Yes	157 (40.99)	307 (39.11)	
No	226 (59.01)	478 (60.89)	
Fetal gender, *n* (%)			0.74
Male	202 (52.74)	406 (51.72)	
Female	181 (47.26)	379 (48.28)	
Gestational age at delivery, weeks	38.45 ± 1.38	38.65 ± 1.60	0.03
Birth weight, g	3282.56 ± 447.82	3287.02 ± 477.40	0.88
Birth height, cm	49.90 ± 1.75	49.82 ± 2.09	0.52

BMI, body mass index; GDM, gestational diabetes mellitus.

a Two-sample student’s *t*-test or Pearson’s chi-squared test or Fisher’s exact test when appropriate.

The mean and standard deviation for serum levels of elements for the cases and controls are shown in Table 2. The levels of serum Zn, Cu, and Ca were significantly higher among cases than among controls (all *p* < 0.05), while the levels of serum Mg, Fe, and Cd in the case group were comparable to those in the control group (all *p* > 0.05). As shown in Supplementary Figure S3A, strong correlations were observed for the serum levels of non-toxic trace elements in pregnant women with GDM. A similar result was also observed in non-GDM (Supplementary Figure S3B).

**Table 2:** Comparison of concentrations of serum trace elements between GDM and the non-GDM pregnancies.

Elements	GDM, *N* = 383^a^	Non-GDM, *N* = 785^a^	*p*-value^b^
Mg (mmol/L)	1.60 ± 0.14	1.59 ± 0.14	0.16
Zn (μmol/L)	124 ± 26	120 ± 26	0.03
Cu (μmol/L)	23.33 ± 1.95	23.03 ± 2.01	0.02
Ca (mmol/L)	1.55 ± 0.11	1.53 ± 0.12	0.02
Fe (μmol/L)	135 ± 28	132 ± 27	0.07
Cd (μg/dl)	0.24 ± 0.09	0.24 ± 0.09	0.24

GDM, gestational diabetes mellitus.

a Data are presented as mean ± SD.

b *p*-values were derived from two-sample student’s *t*-test.

The risk of GDM results in association with each element in crude and single-element models were evaluated (Table 3). Serum Zn, Cu, Ca, and Fe were significantly and positively associated with GDM in crude models (all *p* < 0.05). Except for the element Fe, the other three elements also demonstrated a significant trend in *p*-values. After adjusting the founders, these associations are similar to those in the crude models to a great extent. In the adjusted models, an increased risk of GDM was found for women with second-quartile (OR = 1.48, 95% CI: 1.03–2.13 for Zn; OR = 1.73, 95% CI: 1.20–2.49 for Fe) and fourth-quartile (OR = 1.68, 95% CI: 1.17–2.41 for Zn; OR = 1.57, 95% CI: 1.09–2.26 for Fe) serum Zn and Fe concentrations compared to women with first-quartile concentrations. Serum Cu concentration was still positively associated with the risk of GDM, with ORs of 1.60 (95% CI, 1.12–2.29) and 1.45 (95% CI, 1.01–2.07) for concentrations in the third and fourth quartile, respectively, relative to those in the first quartile. Additionally, the risk of GDM with a fourth-quartile serum Ca concentration was associated with an OR of 1.64 (95% CI, 1.15–2.34) relative to the risk with a first-quartile concentration. The spline analyses suggested a linear association for GDM and Zn, Cu, Ca, and Fe (all *p* > 0.05) but a nonlinear association for Cd (Supplementary Figure S4; *p* = 0.03).

**Table 3:** The association between trace elements and gestational diabetes mellitus using the logistic regression model.

Elements	Case/control	Model 1			Model 2		
		OR (95% CI)^a^	*p*-value	*p*-trend	OR (95% CI)^b^	*p*-value	*p*-trend
Mg				0.11			0.15
Q1	85/214	1.00 (ref.)	—		1.00 (ref.)	—	
Q2	96/194	1.25 (0.88 to 1.77)	0.22		1.26 (0.88 to 1.81)	0.21	
Q3	105/188	1.41 (1.00 to 1.99)	0.05		1.40 (0.98 to 2.01)	0.06	
Q4	97/188	1.30 (0.91 to 1.85)	0.14		1.28 (0.89 to 1.83)	0.19	
Zn				0.02			0.01
Q1	80/211	1.00 (ref.)	—		1.00 (ref.)	—	
Q2	97/195	1.31 (0.92 to 1.87)	0.13		1.48 (1.03 to 2.13)	0.04	
Q3	96/196	1.29 (0.91 to 1.84)	0.16		1.37 (0.95 to 1.98)	0.09	
Q4	109/183	1.57 (1.11 to 2.23)	0.01		1.68 (1.17 to 2.41)	<0.01	
Cu				<0.01			<0.01
Q1	84/211	1.00 (ref.)	—		1.00 (ref.)	—	
Q2	82/211	0.98 (0.68 to 1.40)	0.90		1.03 (0.71 to 1.49)	0.87	
Q3	111/179	1.56 (1.10 to 2.21)	0.01		1.60 (1.12 to 2.29)	0.01	
Q4	105/184	1.43 (1.01 to 2.03)	0.04		1.45 (1.01 to 2.07)	0.04	
Ca				0.02			0.02
Q1	87/223	1.00 (ref.)	—		1.00 (ref.)	—	
Q2	103/207	1.28 (0.91 to 1.80)	0.16		1.36 (0.96 to 1.94)	0.09	
Q3	86/182	1.21 (0.85 to 1.73)	0.29		1.22 (0.85 to 1.77)	0.28	
Q4	106/173	1.57 (1.11 to 2.22)	0.01		1.64 (1.15 to 2.34)	<0.01	
Fe				0.05			0.06
Q1	76/216	1.00 (ref.)	—		1.00 (ref.)	—	
Q2	107/184	1.65 (1.16 to 2.36)	<0.01		1.73 (1.20 to 2.49)	<0.01	
Q3	96/196	1.39 (0.97 to 1.99)	0.07		1.42 (0.98 to 2.06)	0.06	
Q4	103/189	1.55 (1.09 to 2.21)	0.02		1.57 (1.09 to 2.26)	0.02	
Cd				0.19			0.22
Q1	107/221	1.00 (ref.)	—		1.00 (ref.)	—	
Q2	82/198	0.86 (0.60 to 1.21)	0.38		0.87 (0.61 to 1.23)	0.43	
Q3	86/187	0.95 (0.67 to 1.34)	0.77		0.94 (0.66 to 1.34)	0.74	
Q4	107/179	1.23 (0.89 to 1.72)	0.21		1.23 (0.87 to 1.73)	0.23	

a OR, odds ratio; CI, confidence interval; ref., reference.

b Adjusted for maternal age, maternal education, pre-pregnancy BMI and self-reported history of diabetes.

**Figure 1:**
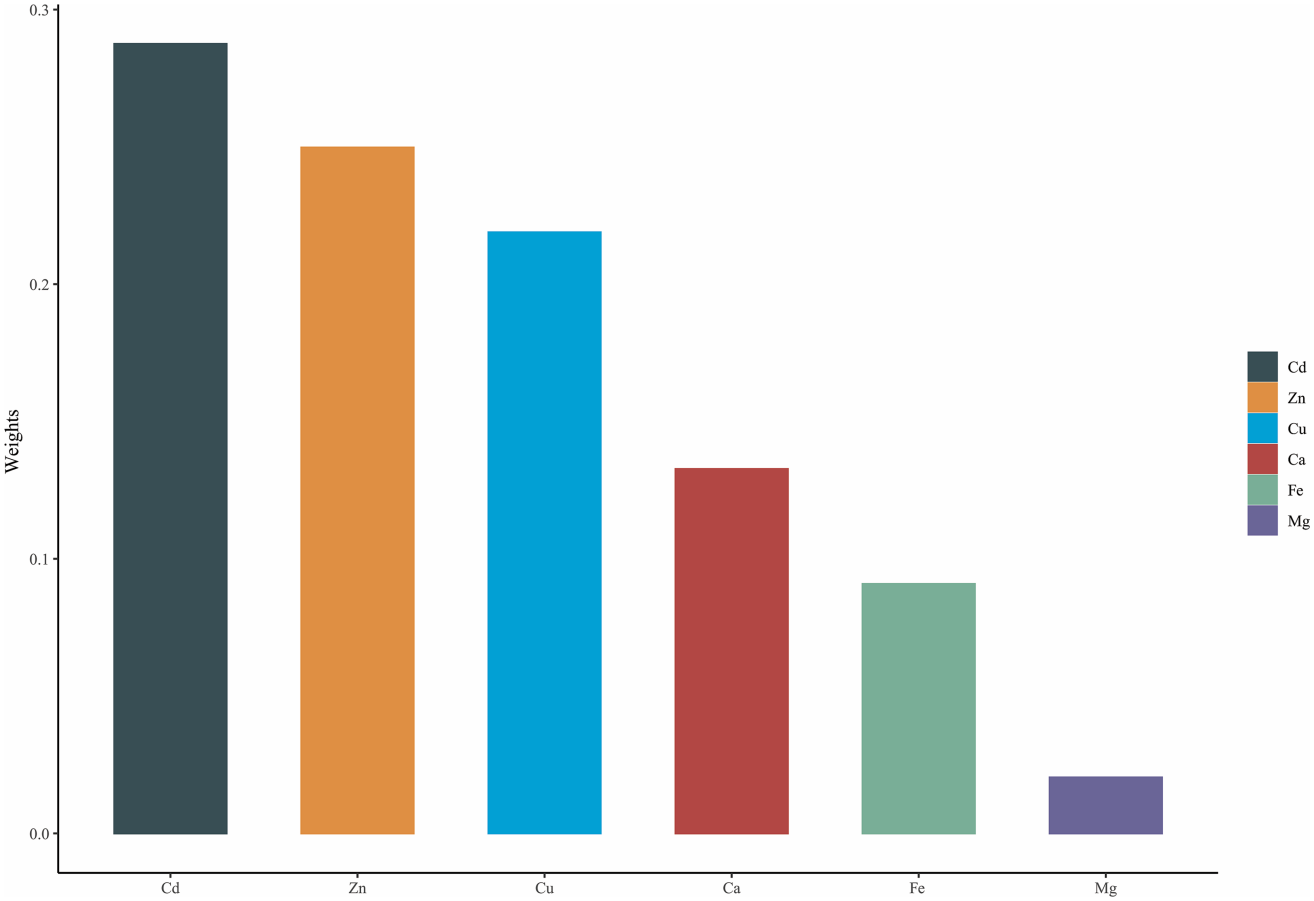
WQS model regression index weights for the gestational diabetes mellitus.

The result derived from WQS regression revealed a significantly positive association between mixed exposure and GDM (OR: 1.38; 95% CI: 1.08–1.76) (Table 4). The major contributor to the element mixture index (WQS index) was Cd (28.75%), followed by Zn (24.97%) and Cu (21.89%) (Figure 1).

**Table 4:** WQS model to estimate the associations between WQS index and gestational diabetes mellitus.

Outcomes	Univariate		Multivariate^b^	
	OR (95% CI)^a^	*p*-value	OR (95% CI)^a^	*p*-value
Positive	1.38 (1.08 to 1.76)	<0.01	1.44 (1.11 to 1.86)	<0.01
Negative	1.22 (0.97 to 1.49)	0.06	1.24 (0.98 to 1.54)	0.06

a OR, odds ratio; CI, confidence interval.

b Adjusted for maternal age, maternal education, pre-pregnancy BMI and self-reported history of diabetes.

**Figure 2:**
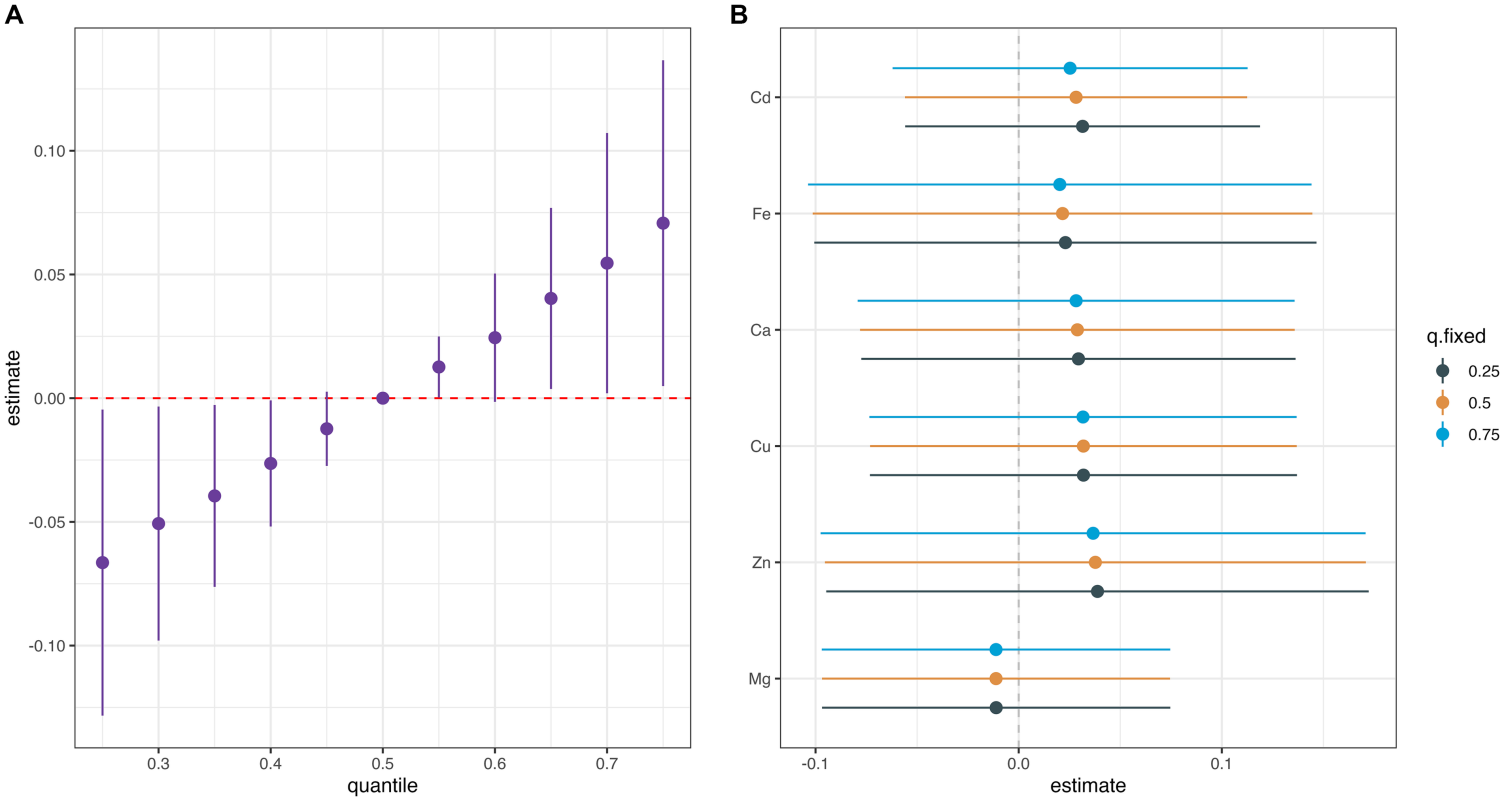
Univariate exposure-response functions (95% CIs) between exposure to single elements and the gestational diabetes mellitus while fixing other metals at their 50th percentiles. **(A)** Overall effect of metal mixture (estimates and 95%CI). **(B)** Single metal association (estimates and 95% CI, estimated zero means null).

**Figure 3:**
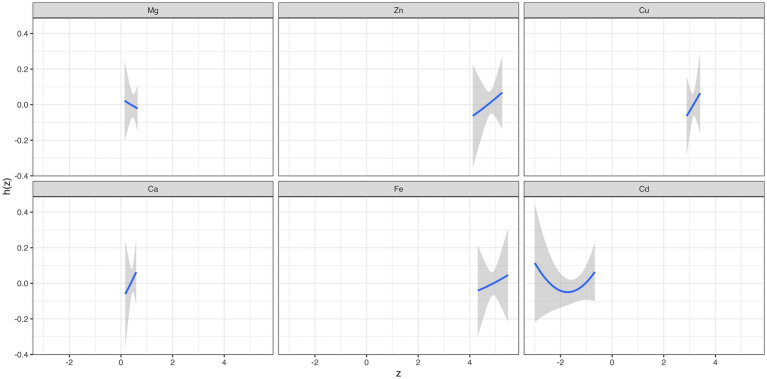
The overall effect and individual effect (estimates and 95% CIs) of serum elements on the risk of gestational diabetes mellitus.

Supplementary Figure S5 summarizes the results of the probabilities of inclusion derived from the BKMR model. The PIP value of Cu (0.56) was the highest, followed by Ca (0.51). The overall associations between the element mixture and GDM are shown in Figure 2A. The serum element mixture at early pregnancy was significantly associated with a higher risk of GDM when all elements were at the 65th percentile or above compared to their 50th percentile, while a decreased risk of GDM was observed when all elements were at the 40th percentile or below. The individual effect of serum levels of six elements on GDM was not significant (Figure 2B). According to the univariate exposure-response associations between a single element and GDM, the dosimetry response curve for Cd tended to be a “U” shape and linear curves were observed for the other elements, which were consistent with the results of spline analyses (Figure 3). No interaction effect was found among the six elements in the bivariate exposure-response analysis, since the slopes of the exposure-response function of a specific element were similar at the different percentiles of other elements, with others fixed at the 50th percentile (Supplementary Figure S6). Moreover, the interaction among six elements was also explored, with no pronounced interaction effect found (Supplementary Figure S7).

Sensitivity analyses were conducted by adjusting all the potential covariates instead of the minimum sufficient set of covariates, and the results were similar (Supplementary Figures S8–S10; Supplementary Table S2). Similar to the WQS model, the quantile g-computation model showed that these six elements were significantly and positively correlated with GDM, both in the unadjusted analysis (*β*: 0.21, 95% CI: 0.04–0.38) and after adjusting for covariates (*β*: 0.22, 95% CI: 0.04–0.39), yielding comparable results.

## 4 Discussion

In this study, we explored the associations between serum levels of six elements (Mg, Cu, Fe, Ca, Zn, and Cd) during the first trimester and the risk of GDM among Chinese pregnant women. The positive relationship of GDM with Zn, Cu, Fe, and Ca was observed in crude and adjusted models. In addition, a significant positive joint effect of the six elements mixture on the GDM risk was found, while Cd, Zn, and Cu appeared to be more critical in the element mixture. However, no significant individual effect of elements on GDM was found in BKMR models.

The concentrations of serum elements included in this study differ from those of several previous studies in China or neighbouring countries. Specifically, the levels of serum Cu, Mg, Fe, Ca, and Zn in GDM were lower than that reported for GDM women in Wuhan determined by inductively coupled plasma mass spectrometry, as well as that of GDM women in Shanghai using inductively coupled plasma-atomic emission spectrometry (17, 36). In contrast, the serum Cd concentrations in healthy pregnant and GDM women (0.24 μg/dL) were comparable to those of occupationally exposed men (2.58 μg/L), but markedly higher than healthy people in previous studies done in China (1.076 μg/L) and Korea (1.34 μg/L) (36). It is worth noting that a wide variation exists in individual element exposure levels across the trimesters (37), and dietary intake and environmental levels of trace elements seemed to vary by geographic region (38, 39). Therefore, the difference in serum element concentrations between our study and previous studies may be partly due to the differences in dietary habits, environmental exposure and detection methods.

In the current study, Zn concentrations in GDM patients were markedly higher than in the non-GDM group. Some (36, 40), but not all (18) cross-sectional studies agreed with our results, revealing that type 2 diabetic or GDM patients have suboptimal Zn status in the blood due to increased urinary depletion. Similarly, inconsistent results were observed among studies with a population-based cohort design (15, 18). The difference in albumin levels might partially explain the above discrepancy to some extent. About 70% of the Zn is bound to albumin and any pathological alteration of albumin affects the blood Zn levels (41). It has been reported that lower serum zinc was observed relating to altered Zn-binding serum proteins in advanced CKD and chronic dialysis patients (42). Normalization of blood Zn levels according to the serum albumin levels should be considered in future studies investigating the relationship between blood Zn and GDM.

It has been speculated that Cu can promote the development of diabetes by continuously providing an active site and increasing oxidative stress, suggesting that Cu may contribute to glucose metabolic disorders through ROS generation (43). Previous studies mainly focused on the difference in Cu concentrations between GDM and the controls, without further exploration of the relation between Cu and GDM risk due to the small sample size (36, 44–46). For example, Wang et al. reported that compared to the control group (*n* = 90), the Cu concentration of the GDM group (*n* = 46) was significantly higher (36). Only a few studies have ventured into this territory. Both case-control and nested case-control studies indicated significantly increased odds of GDM in association with higher concentrations of plasma Cu (17, 47). Recently, a study published in the United States also corroborated these discoveries (16). Our findings regarding the positive relationship between serum Cu level and the risk of GDM are in agreement with previous studies on type 2 diabetes among general populations, offering support to the hypothesis that Cu was involved in the pathogenesis of abnormal glucose metabolism (48).

Ca homeostasis is considered a significant contributory factor in regulating proper insulin secretion and action (49). Currently, most epidemiological research studying the relationship between Ca and glucose homeostasis is based solely on dietary level, whereas limited studies are on the internal exposure level. In contrast to the protective effect of dietary Ca on diabetes (50, 51), a meta-analysis of four studies indicated that both total serum Ca (RR: 1.38, 95% CI: 1.15–1.65) and albumin-corrected Ca (RR:1.29, 95% CI:1.03–1.61) were associated with incident diabetes, which was in line with ours (52). Moreover, a body of growing epidemiologic studies implicated that elevated exposure to Cd promotes the progression of diabetes (12, 22, 23, 53). However, it is noteworthy that only one of these studies delved into the dose-response relationship, yielding non-significant results (53). To the best of our knowledge, our study is the first to report the U-shape effects of serum Cd in the first trimester on GDM. This finding needs further validation.

Even if a single compound is below the safe limits, exposure to multiple compounds may be related to adverse health consequences. In order to determine the joint effects and the possible interactions among elements, we used the BKMR and WQS methods in this pregnant population (32, 33). The results showed a positive combined effect of six elements on GDM. It is noticeable that the association of individual element with GDM risk was attenuated and no longer statistically significant along with the increasing concentrations of other elements in the BKMR model. A relatively strong correlation was observed among the essential elements in the current study, and the trends of these elements with GDM were all positive. Considering the possible overlapping biological functions of essential elements, it may not be surprising to detect the context-dependent nature of the effects of these elements (54). However, other essential elements, such as selenium and manganese, are not measured in this study, which guarantees future research. Further, Zn and Cu were identified to play a more critical role in the risk of GDM under the context of various essential elements, suggesting the mechanism of Zn and Cu involved in the progression of GDM may be, at least partly, independent of other essential elements.

Regarding confounding factors associated with the risk of GDM, maternal age, parity, family history of diabetes and pre-pregnancy BMI were included in the analysis. Notably, we found that the odds of GDM were elevated among primiparous pregnant women, those of advanced age, individuals with a family history of diabetes, and those with a higher pre-pregnancy BMI. These findings align with the conclusions of prior studies. Additionally, our study observed a relatively high proportion of GDM diagnoses, amounting to 32.79%, which is consistent with various reported prevalence rates in different geographic regions of China. It is worth noting that another study, encompassing 690 Chinese pregnant women using the diagnostic criteria of IADPSG, also reported a GDM prevalence of 33.3% (55). The advanced age of the pregnant women in our study, with a mean age of 32.77 and 32.15 years, could potentially account for this higher prevalence. This observation aligns with the conclusions drawn from a meta-analysis, which reported that the incidence of GDM in older Chinese pregnant women could reach as high as 30.3% (3).

The primary strength of our study is that this study is among the few to evaluate prospective associations between specific essential elements at early pregnancy and GDM risk. The prospective design and early measurements of elements minimize the possibility of reverse causation and differential measurement errors. Despite the strengths, several limitations should be noted. First, all the participants included in our study are from Shenzhen, China, which limits the generalizability of our findings to other populations. However, the relative homogeneity of this study population in ethnic background and environmental exposure enhances the internal validity of our findings. Second, elements were measured only in blood samples obtained prior to the 14th week of gestation. While the concentration of elements in blood has been established as a reliable biomarker of exposure, future studies should consider including measurements during the third trimester and at the time of delivery to provide valuable posterior evidence and a more comprehensive understanding of their role in GDM. Third, we failed to measure other forms of serum elements (e.g., ferritin) or key proteins (e.g., albumin) in the current study. Fourth, although various confounding factors like age, pre-pregnancy BMI, gestational age and parity were controlled in our study, there might be other residual confounding that we did not measure but may impact the association examined, such as supplements use during pregnancy. Fifth, limited essential elements and only one toxic metal were included in our study. However, as an exploratory analysis, our study could serve as a valuable starting point for future research endeavors, which may expand the range of elements studied to provide a more comprehensive understanding.

## 5 Conclusion

In conclusion, Zn, Cu, Fe, and Ca exposure in early pregnancy showed a positive association with GDM in the individual evaluation. The multiple evaluations showed that high levels of a mixture of six elements (Zn, Cu, Fe, Ca, Mg, and Cd), particularly Cd, Zn, and Cu, may promote the development of GDM.

## Data availability statement

The raw data supporting the conclusions of this article will be made available by the authors, without undue reservation.

## Ethics statement

The studies involving humans were approved by the Ethics Committee of Huazhong University of Science and Technology Union Shenzhen Hospital (No. 2019072644). The studies were conducted in accordance with the local legislation and institutional requirements. The participants provided their written informed consent to participate in this study.

## Author contributions

GD: Conceptualization, Data curation, Formal analysis, Investigation, Methodology, Software, Writing – original draft, Writing – review & editing. HC: Conceptualization, Data curation, Formal analysis, Investigation, Methodology, Software, Writing – original draft, Writing – review & editing. YL: Investigation, Resources, Writing – review & editing. YZ: Data curation, Investigation, Resources, Writing – review & editing. XL: Data curation, Investigation, Resources, Writing – review & editing. YW: Investigation, Resources, Writing – review & editing. RS: Data curation, Investigation, Resources, Writing – review & editing. ZZ: Conceptualization, Project administration, Writing – review & editing. ZH: Methodology, Project administration, Writing – review & editing.
