# Supplementary material for: Combined exposure to multiple essential elements and cadmium at early pregnancy on gestational diabetes mellitus: a prospective cohort study
Source: Front Nutr. 2023 Dec 6;10:1278617. doi: 10.3389/fnut.2023.1278617 (PMC10730676; doi:10.3389/fnut.2023.1278617)
Supplement: Supplementary file 1 [file Data_Sheet_1.docx]

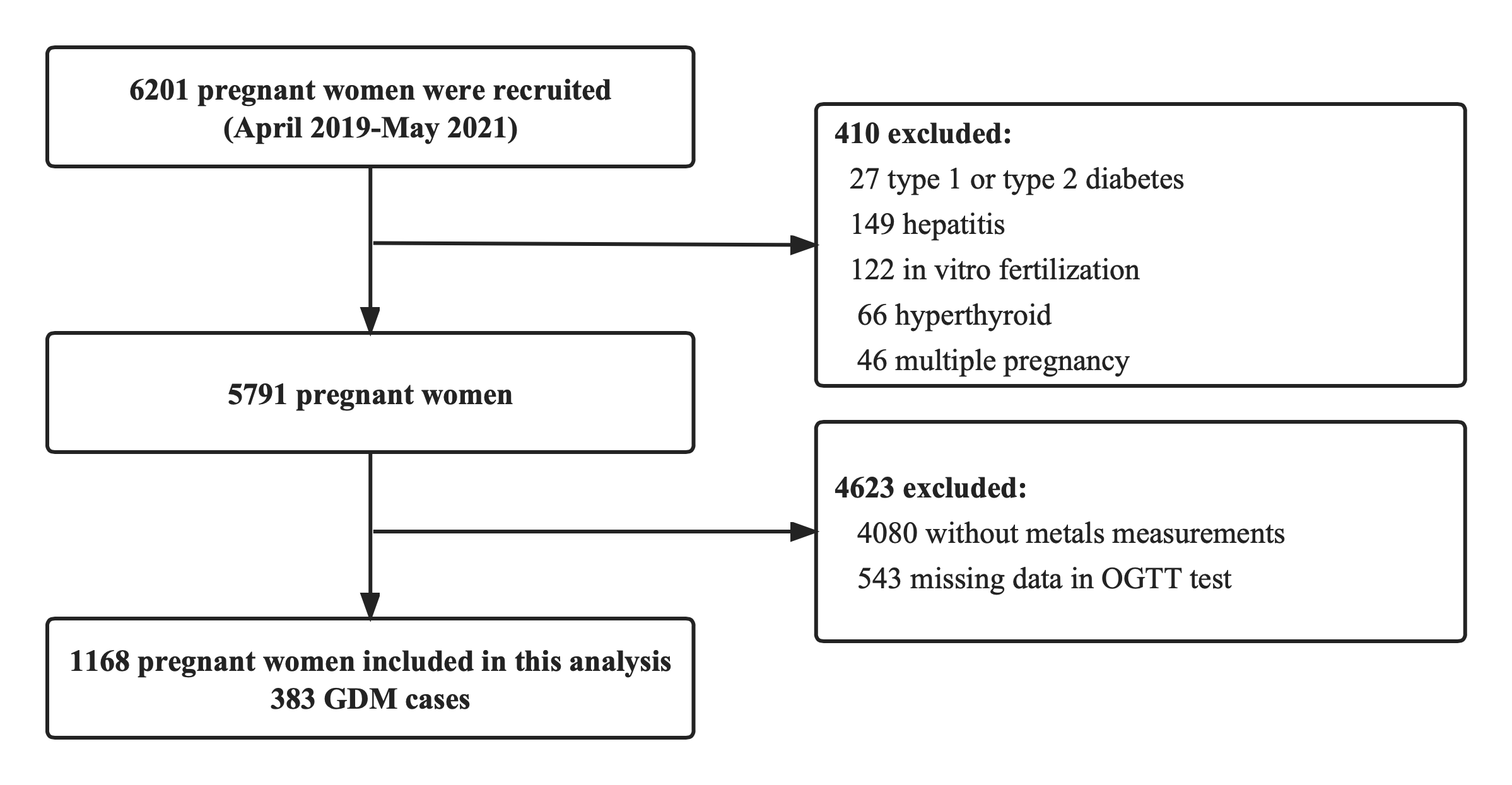


**Fig. S1. Flowchart of study participants**

**Fig. S2. Directed acyclic graph (DAG) for the association between serum elements at the first trimester and gestational diabetes mellitus.**

**Fig. S3. Pearson’s correlation heatmaps among serum concentrations of six elements (Cd, Mg, Fe, Cu, Ca and Zn) in the first trimester among GDM (a) and non-GDM pregnant women (b).**

**
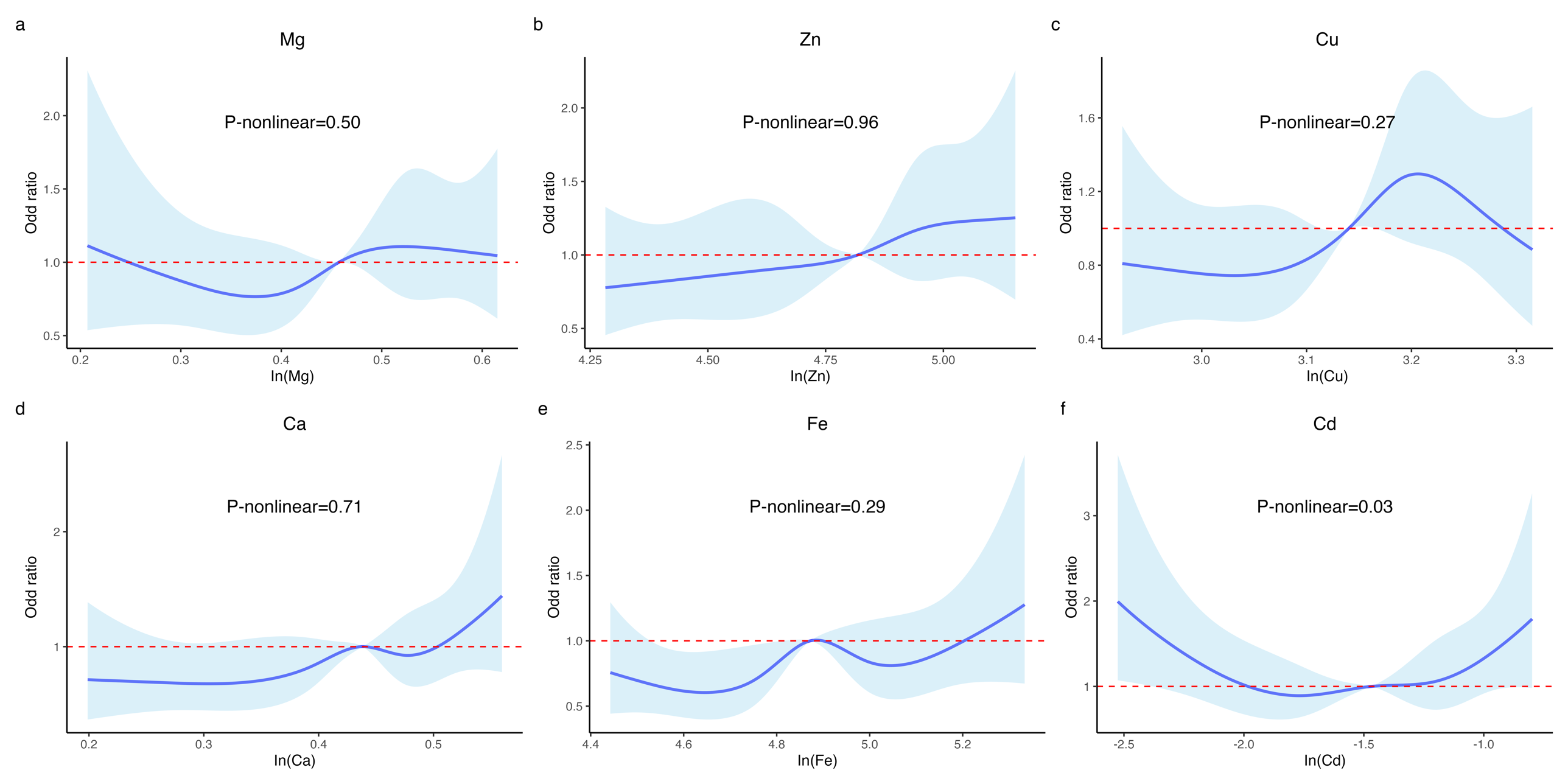
**

**Fig. S4. Nonlinear association between the serum elements at the first trimester and gestational diabetes mellitus using the restricted cubic spline regression. The models were adjusted for maternal age, maternal education, pre-pregnancy BMI and self-reported history of diabetes.**

**Fig. S5. Posterior inclusion probability (PIP) for each of the serum element.**

**

**

**Fig. S6. Bivariate exposure–response functions for: exposure 1 when exposure 2 is fixed at either the 25^th^, 50^th^, or 75^th^ percentiles and other trace elements are fixed at the 50^th^ percentile. The models were adjusted for maternal age, maternal education, pre-pregnancy BMI and self-reported history of diabetes.**

**
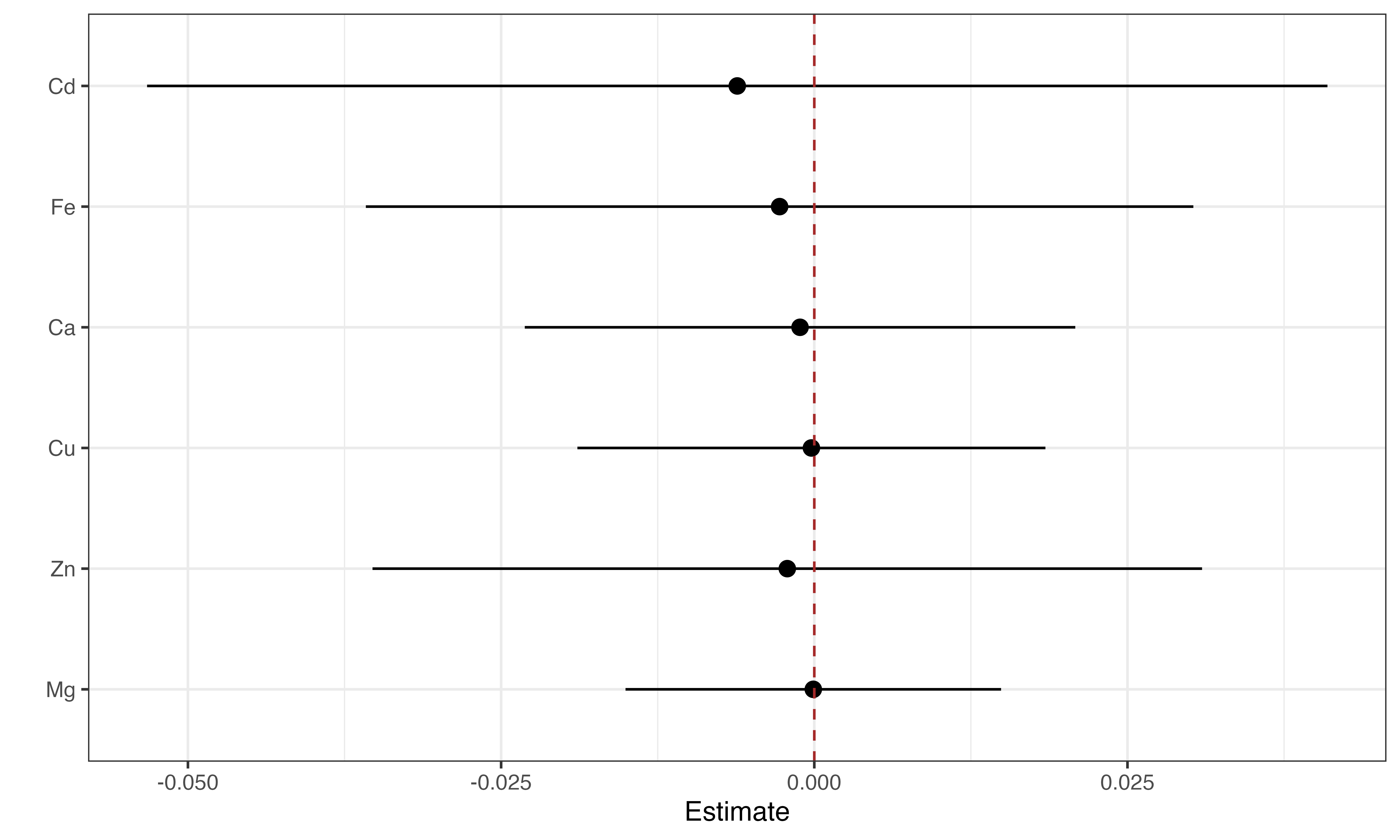
**

**Fig. S7. The interactive effect (estimates and 95% confidence intervals) of six elements. The interactive effect was defined as the change in the individual effect when the concentration of other elements was fixed to their 75^th^ percentile to when their concentration was fixed to their 25^th^ percentile. The model was adjusted for maternal age, maternal education, pre-pregnancy BMI and self-reported history of diabetes**

**Fig. S8. Joint effect of the element mixture on GDM by using Bayesian kernel machine regression adjusted for full set of confounders. (a) Posterior inclusion probability (PIP) for each of the serum element. (b) Overall effect of the element mixture (estimates and 95% confidence intervals). The figure plots the estimated change in a latent continuous outcome when all the elements at particular percentiles (x-axis) were compared to all the elements at their 50th percentile. (c) The estimated values of the individual effect were calculated by comparing the GDM risk when a single element concentration was at its 75th percentile as compared to when that element concentration was at its 25th percentile, where all of the remaining elements concentrations were fixed at their 25th, 50th, or 75th percentile. (d) Univariate exposure-response functions (95% CIs) between exposure to single elements and the gestational diabetes mellitus while fixing other elements at their 50th percentiles. (e) The interactive effect (estimates and 95% confidence intervals) of six elements. (f) Bivariate exposure–response functions for: exposure 1 when exposure 2 is fixed at either the 25^th^, 50^th^, or 75^th^ percentiles and other trace elements are fixed at the 50^th^ percentile.**

**
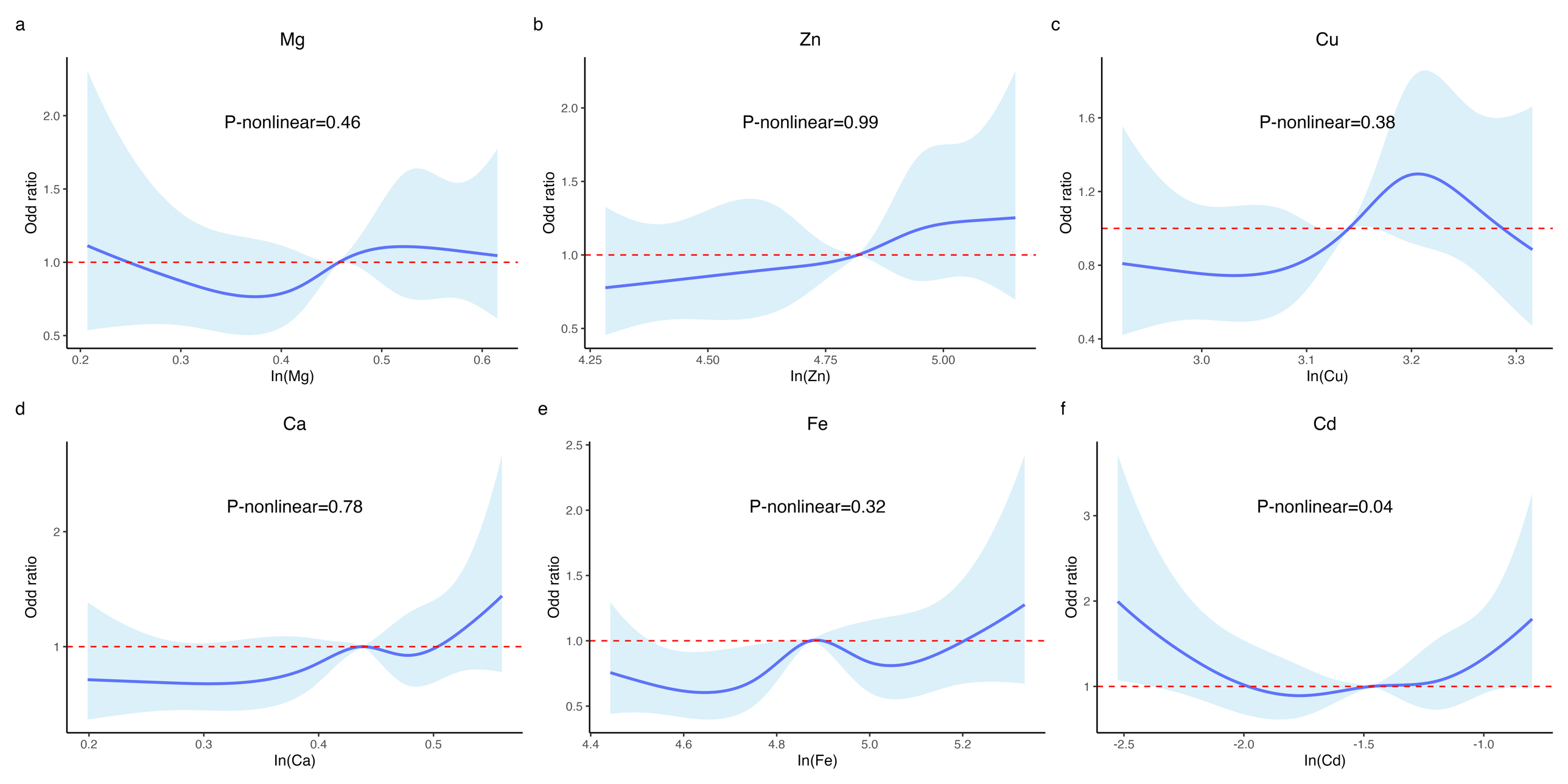
**

**Fig. S9. Nonlinear association between the serum elements at the first trimester and gestational diabetes mellitus using the restricted cubic spline regression adjusted for full set of confounders.**

**Fig. S10. Weighted quantile sum (WQS) regression index weights for GDM estimated in the study population adjusted for full set of confounders. WQS regression model was constrained the association between element mixture exposure and the outcome in the positive direction. WQS model was adjusted for maternal education, maternal age, self-reported history of diabetes, family history of diabetes, gravidity, parity, household registration, history of abortion and fetus gender.**

**Table S1. Maternal characteristics of women who were included and women were excluded in this study.**

| Characteristics | excluded, N = 5033 | included, N = 1168 | *P*-value^1^ |
| --- | --- | --- | --- |
| Maternal age, years | 32.41 ± 4.00 | 32.35 ± 3.93 | 0.70 |
| Pre-pregnancy BMI, kg/m2 | 20.92 ± 3.02 | 21.05 ± 3.51 | 0.25 |
| Household registrations, n (%) |  |  | <0.01 |
| Local resident | 2,957 (58.75) | 792 (67.81) |  |
| Temporary resident | 2,076 (41.25) | 376 (32.19) |  |
| Maternal education, n (%) |  |  | <0.01 |
| Junior middle school and below | 316 (6.28) | 42 (3.60) |  |
| High school | 2,148 (42.68) | 454 (38.87) |  |
| University and above | 2,569 (51.04) | 672 (57.53) |  |
| Family history of diabetes, n (%) |  |  | 0.83 |
| Yes | 28 (0.56) | 7 (0.60) |  |
| No | 5005 (99.54) | 1161 (99.40) |  |
| Self-reported history of diabetes, n (%) |  |  | 0.11 |
| Yes | 37 (0.74) | 14 (1.20) |  |
| No | 4996 (99.26) | 1154 (98.80) |  |
| Gravidity, n (%) |  |  | 0.63 |
| 1 | 1270 (25.23) | 303 (25.94) |  |
| ≥2 | 3763 (74.77) | 865 (74.06) |  |
| Unknown |  |  |  |
| Parity, n (%) |  |  | 0.74 |
| 1 | 2000 (39.71) | 470 (40.24) |  |
| ≥2 | 3033 (60.29) | 698 (59.76) |  |
| History of abortion, n (%) |  |  | 0.55 |
| Yes | 1952 (38.78) | 464 (39.73) |  |
| No | 3081 (61.22) | 704 (60.27) |  |
| GDM, n (%) |  |  | 0.15 |
| GDM | 1535 (30.50) | 383 (32.79) |  |
| non-GDM | 3498 (70.97) | 785 (67.21) |  |
| Fetal gender, n (%) |  |  | 0.56 |
| Male | 2570 (51.06) | 608 (52.05) |  |
| Female | 2463 (48.94) | 560 (47.95) |  |
| Gestational age at delivery, weeks | 38.67 ± 1.59 | 38.58 ± 1.46 | 0.08 |
| Birth weight, g | 3284.18 ± 472.90 | 3284.02 ± 457.53 | 0.98 |
| Birth height, cm | 49.85 ± 1.89 | 49.87 ± 1.87 | 0.71 |
| ^1^Welch Two Sample t-test; Pearson's Chi-squared test; Fisher's exact test | | | |

**Table S2. The association between trace elements and gestational diabetes mellitus using the logistic regression adjusting for full set of confounders.**

| Elements | Q1 | Q2 | |  | Q3 | |  | Q4 | | *P*-trend |
| --- | --- | --- | --- | --- | --- | --- | --- | --- | --- | --- |
|  |  | OR^1^ | 95% CI^1^ |  | OR^1^ | 95% CI^1^ |  | OR^1^ | 95% CI^1^ |  |
| Mg | ref. | 1.26 | 0.88, 1.82 |  | 1.37 | 0.96, 1.97 |  | 1.25 | 0.87, 1.81 | 0.20 |
| Zn | ref. | 1.39 | 0.97, 2.02 |  | 1.36 | 0.94, 1.96 |  | **1.60** | **1.12, 2.31** | 0.02 |
| Cu | ref. | 1.01 | 0.69, 1.46 |  | **1.54** | **1.07, 2.20** |  | **1.41** | **1.02, 2.02** | 0.01 |
| Ca | ref. | 1.31 | 0.92, 1.87 |  | 1.22 | 0.84, 1.76 |  | **1.57** | **1.10, 2.25** | 0.03 |
| Fe | ref. | **1.71** | **1.19, 2.47** |  | **1.35** | **0.93, 1.96** |  | **1.53** | **1.06, 2.21** | 0.10 |
| Cd | ref. | 0.88 | 0.62, 1.26 |  | 0.99 | 0.69, 1.42 |  | 1.31 | 0.93, 1.84 | 0.11 |

^1^OR: odd ratio; CI: confidential interval.

The models were adjusted for maternal education, maternal age, self-reported history of diabetes, family history of diabetes, gravidity, parity, household registration, history of abortion and fetus gender.

**Table S2. The joint association between trace elements and gestational diabetes mellitus using the quantile g-computation for minimal set of confounders.**

| Models | β | 95% CI |
| --- | --- | --- |
| Crude model | 0.21 | 0.04, 0.38 |
| Adjusted model^1^ | 0.22 | 0.04, 0.39 |

^1^: adjusted for maternal age, maternal education, pre-pregnancy BMI and self-reported history of diabetes.
